# Carnivorous diving beetles of the genus *Desmopachria* (Coleoptera: Dytiscidae) from Brazil: New species, new records, and a checklist

**DOI:** 10.1093/jis/14.1.55

**Published:** 2014-01-01

**Authors:** Rafael Benzi Braga, Nelson Ferreira

**Affiliations:** 1 Laboratório de Entomologia, Departamento de Zoologia, Instituto de Biologia, Universidade Federal do Rio de Janeiro, Caixa Postal 68044, Rio de Janeiro, RJ, 21941-971, Brasil

**Keywords:** catalog, description, distribution, Neotropic, taxonomy

## Abstract

Eight new species of *Desmopachria*Babington, 1841 are described and illustrated from Brazil: *D. dicrophallica*** sp. nov.** , *D. disticta*** sp. nov.** , *D. grammosticta*** sp. nov.** , *D* . *grandinigra*** sp. nov.** , *D. itamontensis*** sp. nov.** , *D. leptophallica*** sp. nov.** , *D. stethothrix*** sp. nov.** , and *D. ukuki*** sp. nov.** The species *D. amyae*Miller, 2001 , *D. chei*Miller, 1999 , *D. margarita* Young, 1990, and *D. volatidisca*Miller, 2001 are recorded for the first time from Brazil. From species of the *Desmopachria* reported in Brazil, *D. aldessa*Young, 1980 has a new record from Pará state and *D. fossulata*Zimmermann, 1928*, D. granoides*Young, 1986 , and *D. laevis* Sharp, 1882 have new records from Rio de Janeiro State. A checklist of all *Desmopachria* recorded from Brazil is presented with notes about some of the localities.

Resumo

Oito espécies novas de *Desmopachria*Babington, 1841 são descritas e ilustradas para o Brasil, *D. dicrophallica*** sp. nov.** , *D. disticta*** sp. nov.** , *D. grammosticta*** sp. nov.** , *D* . *grandinigra* sp. nov., *D. itamontensis* sp. nov., *D. leptophallica* sp. nov., *D. stethothrix*** sp. nov.** e *D. ukuki*** sp. nov.** As espécies *D. amyae*Miller, 2001*, D. chei*Miller, 1999 , *D. margarita* Young, 1990 e *D. volatidisca*Miller, 2001 são registradas pela primeira vez para o Brasil. Das espécies de *Desmopachria* registradas para o Brasil *D. aldessa*Young, 1980 tem um novo registro para o estado do Pará e *D. fossulata*Zimmermann, 1928 , *D. granoides*Young, 1986 e *D. laevis* Sharp, 1882 têm novos registros para o estado do Rio de Janeiro. Uma listagem de todos os *Desmopachria* registrados para o Brasil é apresentada, com notas acerca de algumas localidades.

## Introduction

The genus *Desmopachria*Babington, 1841 is one of the largest Dytiscidae groups around the world, with 111 valid species at the present ( Braga and Ferreira-Jr 2011 ; Makhan 2012 ; Megnar and Sánchez- Fernández 2014 ), 58 of which are recorded from Brazil. These species are common in Neotropic and southern Nearctic freshwater areas and occur in a variety of lentic habitats, including ponds, streams, forest pools, bromeliads, and tree hole pools ( Miller 2005 ).

*Desmopachria* can be collected in large numbers using black lights and mercury vapor lamps ( Miller 2005 ). Due to the small size of the insects, manual collections or collections using sieves rarely obtain these species. However, because not all species are attracted to light traps, the most appropriate way to manually collect these small predaceous diving beetles is to use samplers with mesh of 1 mm in diameter or less. However, care must be taken during collection because these samplers tend to get coated with sediment very quickly. A very effective method is to place the sampler over a plastic tray immediately after scraping. The water drained through the mesh usually contains small aquatic beetles, which are easily seen in the bottom of the tray. The compression of the sediment and of the vegetation from the banks of aquatic environments allowing water runoff to a plastic tray usually brings along some small aquatic beetles. Despite difficulties of collection, new species of the *Desmopachria* are regularly encountered, and it seems likely that the true number of species is significantly higher than currently known.

*Desmopachria* are small (1.00–2.50 mm in length), rounded beetles with colors varying from light brown to black, and they may have spots or streaks. The beetles are usually without sexual dimorphism. Miller (2001) listed a series of diagnostic characters of this genus, such as reduction of the metacoxal lobes, reduction of the anterior metatarsal claw, antennomeres 1–2 wider than following articles, antennomeres 5–10 short and slightly expanded in apical half, labial palpus with apical pair of sensilla widely separated, maxillary palpus with an apical sensillum, pronotum with posterolateral angles produced backward, metacoxae fused to visible abdominal sternite one, and metatibia with apical transverse row of spines discontinuous medially.

In our study, eight new species of *Desmopachria* from Brazil are described and illustrated in this article, and new records for *D. amyae*Miller, 2001 , *D. chei*Miller, 2001 , *D. margarita* Young, 1990 and *D. volatidisca*Miller, 2001 from Brazil are presented *.* From species of the *Desmopachria* reported in Brazil, *D. aldessa*Young, 1980 has a new record from Pará State and *D. fossulata*Zimmermann, 1928 , *D. granoides*Young, 1986 , and *D. laevis* Sharp, 1882 have new records from Rio de Janeiro State. A checklist of all *Desmopachria* recorded from Brazil is presented with notes about some localities.

## Materials and Methods

The specimens were examined under a stereoscopic microscope with up to 150 times magnification. The measurements were obtained with the aid of a grid ocular with accuracy of 0.01 mm onto a stereomicroscope, and specimens were preserved in tubes with ethyl alcohol 92%. The genitalia were mounted in slides and coverslips with glycerin gel to drawings and stored in microvials within the specimen tube. Because the species of this genera usually lack external sexual dimorphism, the only way of determinate the sex of specimens is examining the genitalia, therefore only the sex of the holotype was determined. The terminology used in the descriptions follows that of Young (1980 , 1995) and Miller (2001 , 2005). The specimens were deposited in Coleção Entomológica Prof. José Alfredo Pinheiro Dutra, Departamento de Zoologia, Universidade Federal do Rio de Janeiro, Brazil (DZRJ) and in the entomological collections of the Instituto Nacional de Pesquisas da Amazônia (INPA) and Museu de Zoologia da Universidade de São Paulo (MZSP). The specimens from this work were collected under the federal license (IBAMA) numbers 002/2004, 001/2005, 003/2006, 012/2006, 008/2007 MAB/FAUNA; 124/2004, 289/05, 321/2006 MMA/IBAMA; 11081-1, 14591-1, 14591-2, 14591-3, 14591-4 MMA/ICMBIO; 11081-1, 19206-1, MMA/IBAMA, ICMBIO.

In the descriptions of new species, the collection were the specimens were deposited is given first, the geographic coordinates are listed second, and the labels accompanying the specimens are listed third, each between quotation marks and separated by bars. When the geographic coordinates of a collection place were unknown, the central point of the municipality is presented. A checklist of all *Desmopachria* reported from Brazil is presented, with the locations and their authors in parentheses. In the list, we denote new records by (*). Notes explaining common location changes are presented before the list, and explanations for specific cases within the list are below the referred species.

### Nomenclature

This publication and the nomenclature it contains have been registered in Zoobank. The LSID number is:

urn:lsid:zoobank.org:pub:4EF7A35B-0535-4FC8-AB3B-BC1E3A88AF0B

It can be found online by inserting the LSID number after www.zoobank.org/.

*Desmopachria dicrophallica* **sp. nov.**

( Figures 1–3 )

**Figures 1–3.**
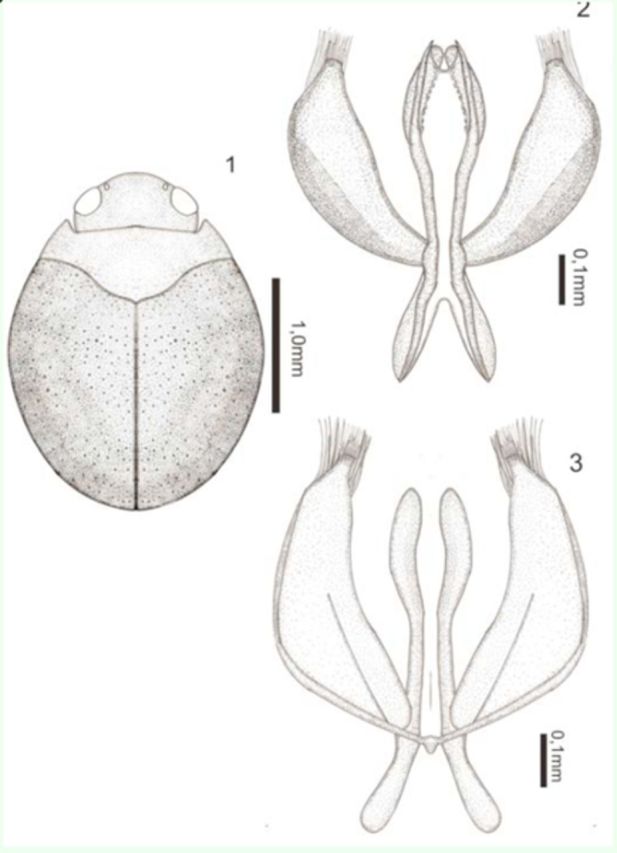
*Desmopachria dicrophallica* **sp. nov.** 1) Habitus, dorsal. 2) Male genitalia, ventral view. 3) Male genitalia, dorsal view. High quality figures are available online.

**Description.** Holotype male: total length 2.30 mm; maximum width 1.50 mm; elytral length 1.60 mm; maximum width of the pronotum 1.10 mm. Head, pronotum, and ventral surface light brown; elytron dark brown ( Figure 1 ).

Body rounded. Head inconspicuously punctate; clypeus lightly truncate and beaded; antennomeres 6–10 slightly expanded in apical half. Pronotum short and wide, very fine and inconspicuously punctuate except for the posterior margin, where the punctures are coarse, without basal striae, and lateral bead wide. Prosternal process apically strongly forked, area between rami forming a deep pit; metasternum, metacoxae, and ventrites without punctures. Elytron with coarse punctures. Aedeagus strongly margined, with base wide narrowing to apex and subtly widening in the apical third. Paramere thin and broad with a narrowing in the apex, apex rounded and surrounded with dense rows of setae in both sides ( Figures 2–3 ).

**Intraspecific variation.** Total length 2.10– 2.30 mm; maximum width 1.40–1.50 mm; elytral length 1.40–1.60 mm; maximum width of the pronotum 1.00–1.10 mm.

**Type material.** Holotype: male (DZRJ Coleoptera 3162) (06°06'14.08''S 50°08'13.01''W), “Brasil, PA, Parauapebas, Flona Carajás, Serra Norte, Buritizal 1, (Riacho de 3º e 4º ordem), 16.ix.2006, Ferreira-Jr N. and Dumas L.L.” / “Holotipo” [plastic red label] / “3162” [plastic red labels]. Paratypes: 7 exs (DZRJ Coleoptera 3163) (06º04'13.32''S 49°56'59.63''W), “Brasil, PA, Parauapebas, Carajás, Buritizal II, poça com folhiço, 08.ix.2006, Ferreira-Jr N. and Dumas L.L.” / “Paratipo” [plastic red label] / “3163” [plastic black label].

**Etymology.** The specific epithet is the Greek adjective **dikros,** meaning “forked,” and the Greek noun **phallos** , meaning “aedeagus,” referring to the shape of aedeagus.

**Taxonomic notes.** This new species belongs to the *D. portmanni* group **sensu**Miller (2001) in having the prosternal process sexually dimorphic, male process apically strongly forked, area between rami forming a deep pit, female process not as in male. The shape of genitalia this species is unique. The dilatation in third apical, the jagged and surrounded with rows of setae in both sides are unprecedented features in this genus.

*Desmopachria disticta* **sp. nov.**

( Figures 4–6 )

**Figures 4–6.**
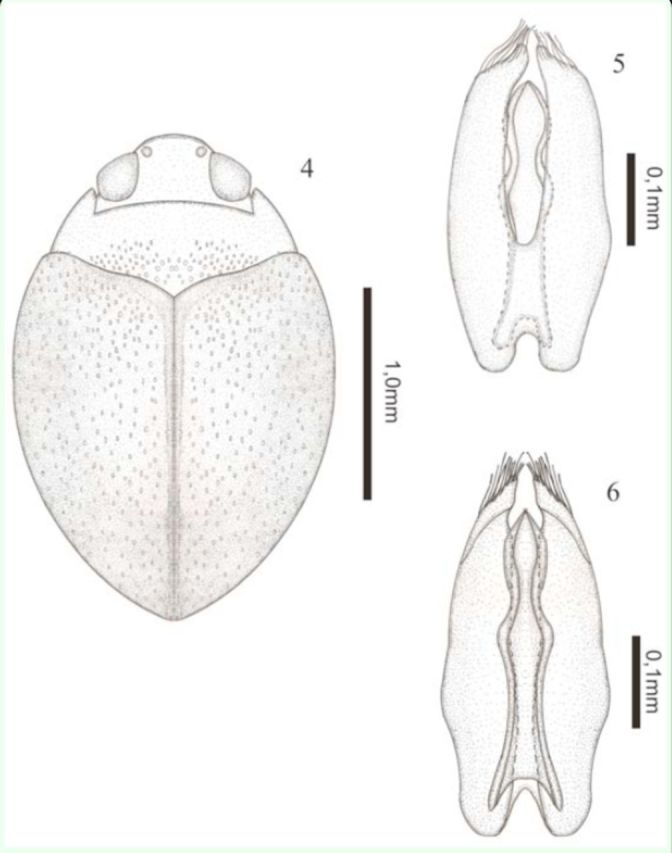
*Desmopachria disticta* **sp. nov.** 4) Habitus, dorsal. 5) Male genitalia, ventral view. 6) Male genitalia, dorsal view *.* High quality figures are available online.

**Description.** Holotype male: total length 2.13 mm; maximum width 1.45 mm; elytral length 1.40 mm; maximum width of the pronotum 1.25 mm. Head, pronotum, and ventral surface light brown; elytron dark brown ( Figure 4 ).

Body rounded. Head inconspicuously punctate; clypeus inconspicuously truncate and not beaded; antennomeres 8–10 slightly expanded in apical half. Pronotum short and wide, very fine and inconspicuously punctuate except for center of base, where punctures are coarse, without basal striae, and lateral bead wide; prosternal process apically strongly forked, area between rami forming a deep pit; metasternum, metacoxae, and abdomen with printed lines short and irregular, without punctures. Elytron with two types of punctures, a very fine and inconspicuous and others coarse. Aedeagus margined with forked base, laterally expanding into center apex; apex acute. Paramere broad, narrowing subtly near apex, with rows of setae in outer margin; apical area flattened, in dorsal view ( Figures 5–6 ).

**Intraspecific variation.** Body measurements vary in total length from 2.10 to 2.20 mm; maximum width 1.45–1.48 mm; elytral length 1.38–1.50 mm; maximum width of pronotum 1.13–1.25 mm.

**Type material.** Holotype: male (INPA) (00°51'57.82''N 63°28'01.99''W); “Brasil, AM, Barcelos, Serra do Aracá, poça com folhiço, (B02), 23.vii.2009, Ferreira-Jr N.” / “Holotipo” [plastic red label]; Paratypes: 5 exs (INPA) (00°52'35.33''N 63°28'01.78''W), “Brasil, AM, Barcelos, Serra do Aracá, poças no caminho para o Igarapé do Tatu, B05, 04.viii.2009, Ferreira-Jr N.” / “Paratipo” [plastic red label]; 1 ex. (INPA) (00°52'47.78''N 63°28'27.05''W), “BR, AM - Barcelos, Igarapé do Jabuti, 04.viii.2009, Folhiço Remanso, Ferreira-Jr N.” / “Paratipo” [plastic red label]; 2 exs. (INPA) (00°52'34.21''N 63°28'27.05''W), “Brasil, AM, Serra do Aracá, Igarapé da Cobra, poças de pedra, B08, 06.viii.2009, 00,87617oN 63,45100oW, Santos A.P.M. & Neiss U.” / “Paratipo” [plastic red label]; 2 exs (INPA) (00°52'35.33''N 63°28'1.78''W), “Brasil, AM, Barcelos, Serra do Aracá, Igarapé do Tatu, 24.viii.2009, B04, Poças na margem, Ferreira-Jr N.” / “Paratipo” [plastic red label]; 3 exs (DZRJ Coleoptera 2957) (00°52'34.21''N 63°28'27.05''W), “Brasil, AM, Barcelos, Serra do Aracá, Igarapé da Cobra, 29.viii.2009, B08, Poças em pedra, Ferrreira-Jr N.” / “Paratipo” [plastic red label] / “2957” [plastic black label]; 5 exs (DZRJ Coleoptera 2958) (00°51'57.82''N 63°28'01.99''W), “Brasil, AM, Barcelos, Serra do Aracá, ponto B02, poças com fohiço,00,86606oN 63,46722oW, 23.viii.2009, Ferreira-Jr N.” / “Paratipo” [plastic red label] / “2958” [plastic black label]; 5 exs (DZRJ Coleoptera 2959) (00°45'25.60''N 63°26'26.02''W), “Brasil, AM, Barcelos, acampamento base, 05.viii.2009, poça com folhiço, B808, Ferreira-Jr N.” / “Paratipo” [plastic red label] / “2959” [plastic black label].

**Etymology.** The specific epithet is the Greek prefix **di** meaning “two,” and the Greek adjective **stiktos** , meaning “punctured,” referring to double punctures on the dorsal surfaces of the pronotum and elytra.

**Taxonomic notes.** This new species belongs to the *D. portmanni* group **sensu**Miller (2001) in having the prosternal process sexually dimorphic, male process apically strongly forked, area between rami forming a deep pit, female process not as in male. Different types of punctures on pronotum also are present in *Desmopachria bryanstonii*Clark, 1862 , *D. decorosa*Young, 1995 , *D. dispar* Sharp, 1882, and *D. laevis* , but in none of these species is it so abrupt and conspicuous. The genitalia of *D. disticta*** sp. nov.** is not typical of the *D. portmanni* group, with lateral expansions of aedeagus and parameres wide with a row of setae on the outer margin instead of the inner.

**Geographic distribution** . So far known only from the Amazonas State in Brazil.

*Desmopachria grammosticta* **sp. nov.**

( Figures 7–9 )

**Figures 7–9.**
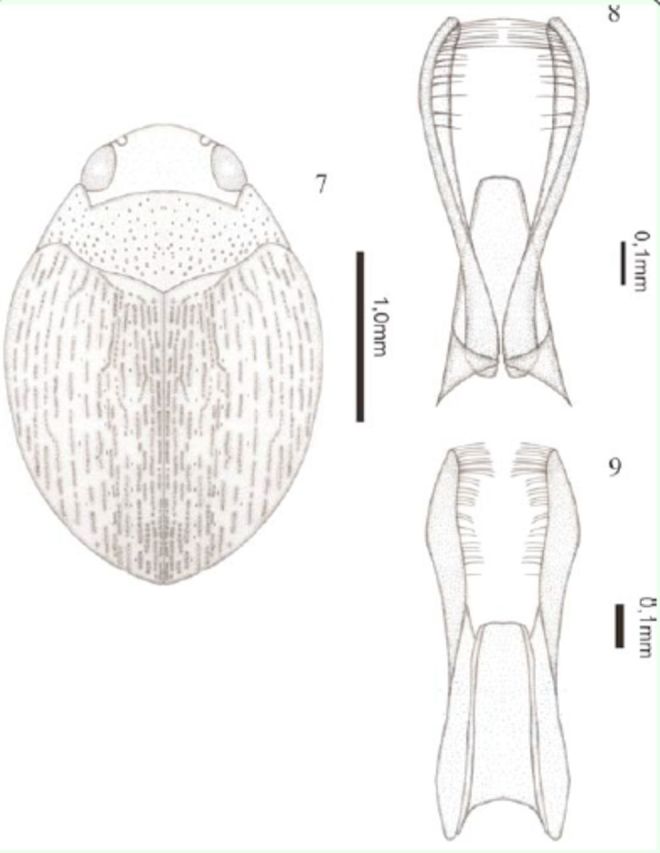
*Desmopachria grammosticta* **sp. nov.** 7) Habitus, dorsal. 8) Male genitalia, ventral view. 9) Male genitalia, dorsal view. High quality figures are available online.

**Description.** Holotype male: total length 2.52 mm; maximum width 1.80 mm; elytral length 1.65 mm; maximum width of the pronotum 1.29 mm. Dorsal and ventral surface dark brown ( Figure 7 ).

Body rounded. Head inconspicuously punctate; clypeus indistinctly beaded; antennomeres 6–10 slightly expanded in apical half. Pronotum short and wide, with two types of punctures, a very fine and others coarse, without basal striae and lateral bead; prosternal process apically strongly forked, area between rami forming a deep pit. Metasternum, metacoxae, and abdomen with coarse punctures. Elytron with two types of punctures, a scattered very fine and others dark and very coarse forming rows. Aedeagus with half of length of paramere, margined with base wide narrowing to apex; apex rounded. Paramere thin, distal third with internal edge flattened, and internal margin with rows of setae ( Figures 7–9 ).

**Intraspecific variation.** Body measurements vary in total length from 2.40 to 2.52 mm; maximum width 1.65–1.80 mm; elytral length 1.62–1.80 mm; maximum width of pronotum 1.26–1.38 mm.

**Type material.** Holotype: male (INPA) (00°52'34.21''N 63°28'27.05''W), “Brasil, AM, Barcelos, Acampamento Base, poça com folhiço, (B808) 5.viii.2009, Ferreira-Jr N.” / “Holotipo” [plastic red label]; Paratypes: 18 exs. (INPA) (00°52'34.21''N 63°27'56.70''W), “Brasil, AM, Barcelos, poças no caminho para Igarapé do Tatu, B05, 04.viii.2009, Ferreira-Jr N.” / “Paratipo” [plastic red label]); 1 ex. (INPA) (00°24'33.88''N 63°23'17.16''W), “Brasil, AM, Barcelos, Serrinha, Rio Aracá, 06.viii.2009, Kinon, Ferreira-Jr N.” / “Paratipo” [plastic red label]; 4 exs. (INPA) (00°51'57.82''N 63°28'01.99''W), “Brasil, AM, Barcelos, Serra do Aracá, ponto B02, poças com folhiço, 00,86606oN 63,467220oW, 23.viii.2009, Ferreira-Jr N.” / “Paratipo” [plastic red label]; 2 exs. (INPA) (00°52'24.96''N 63°27'19.15''W), “Brasil, AM, Barcelos, Serra do Aracá, acampamento base, B01, 25.viii.2009, Panelas com água, Santos A.P.M.” / “Paratipo” [plastic red label]; 37 exs. (DZRJ Coleoptera 2960) (00°52'34.21''S 63°28'27.05''W), “BR, AM, Barcelos, Serra do Aracá, Igarapé da Cobra, 9.viii.2009, Poças na mata, Ferreira-Jr N.” / “Paratipo” [plastic red label] / “2960” [plastic blue label]; 2 exs. (INPA) (00°52'35.33''N 63°28'01.78''W), “Brasil, AM, Barcelos municipality, Igarapé do Tatu, 24.viii.2009, Ferreira-Jr N.” / “Paratipos” [plastic red label]; 2 exs. (DZRJ Coleoptera 2961) (00°52'34.21''N 63°27'56.70''W), “Brasil, AM, Barcelos, Serra do Aracá, Igarapé do Tatu, B05, 24.viii.2009, poças na margem, Ferreira-Jr N.” / “Paratipo” [plastic red label] / “2961” [plastic black label]; 1 ex. (DZRJ Coleoptera 2962) (00°52'34.21''N 63°28'27.05''W), “Brasil, AM, Barcelos, Serra do Aracá, Igarapé da Cobra, Poças na pedra, B08, 06.viii.2009, 00,97617oN 63,4511oW, Santos A.P. & Neiss U.” / “Paratipo” [plastic red label] / “2962” [plastic black label]; 4 exs. (DZRJ Coleoptera 2963) (00°52'34.21''N 63°28'27.05''W), “Brasil, AM - Barcelos, Serra do Aracá, Igarapé da Cobra, B08, 29.viii.2009, poças na pedra, Ferreira-Jr N.” / “Paratipo” [plastic red label] / “2963” [plastic black label]; 1 ex. (DZRJ Coleoptera 2964) (00°52'34.21''N 63°28'27.05''W), “Brasil, AM, Barcelos, acampamento base, bainha de palmeira, B08, 05.viii.2009, Ferreira-Jr N” / “Paratipo” [plastic red label] / “2964” [plastic black label]; 10 exs. (DZRJ Coleoptera 2965) (00°52'34.50''N 63°27'56.20''W), “AM, Barcelos, Serra do Aracá, poças - B10,30.viii.2009, Nessimian J.L., Santos J.O. & Neiss U.G.” / “Paratipo” [plastic red label] / “2965” [plastic black label].

**Etymology.** The specific epithet is the Greek prefix **gramme,** meaning “line,” and the Greek adjetive **stiktos** , meaning “punctured,” referring to the linear rows formed by coarse punctures on the elytral surface.

**Taxonomic notes.** This new species belongs to the *D. portmanni* group **sensu**Miller (2001) in having the prosternal process sexually dimorphic, male process apically strongly forked, area between rami forming a deep pit, female process not as in male, and the characteristic genitalia with paramere curved, with a row of bristles on the internal edge. The coarse dark punctures forming rows in the elytron occurs in *D. goias*Young, 1995 and *D. variegata* Sharp, 1882, however both species are much smaller in size and the length of aedeagus is bigger. The genitalia are only comparable to *D. aurea*Young, 1980 , but the aedeagus base of this species is thinner than *D. grammosticta*** sp. nov.,** and *D. aurea* is smaller in sizeand with a different colour pattern.

**Geographic distribution** : So far known only from the Amazonas State in Brazil.

*Desmopachria grandinigra* **sp. nov.**

( Figures 10–12 )

**Figures 10–12.**
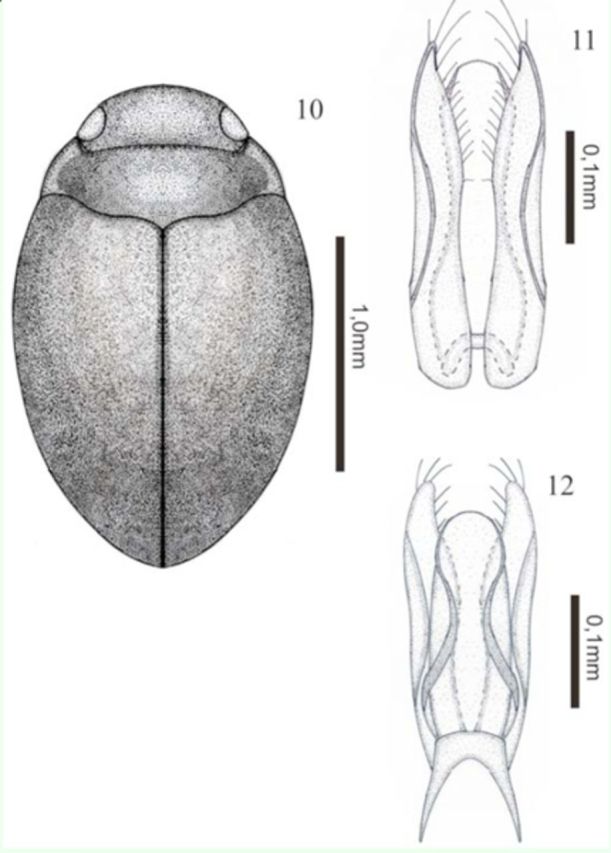
*Desmopachria grandinigra* **sp. nov.** 10) Habitus, dorsal. 11) Male genitalia, ventral view. 12) Male genitalia, dorsal view. High quality figures are available online.

**Description.** Holotype male: total length 2.25 mm; maximum width 1.41 mm; elytral length 1.59 mm; maximum width of the pronotum 1.17 mm. Dorsal surface of head, elytra, pro-, meso- and metasternum, metacoxae, and abdomem shining black; pronotum shining black, reddish in lateral margin ( Figure 10 ); antennae, palps, and legs reddish.

Body rounded, very fine, and inconspicuously punctate. Clypeus indistinctly beaded; antennae with rounded scape, pedicel narrower and longer than flagellomeres, antennomeres 5–9 slightly expanded in apical half. Pronotum short and wide; basal striae and lateral beads absent. Pro- and mesotarsum with ventral adhesive setae; prosternal process apically strongly forked, area between rami forming a deep pit. Ventral surface of metatibia covered with short and wide spines. Aedeagus with wave margin; base bifurcates and apex rounded. Paramere thin, with wave margin and row of setae in internal margin before apex ( Figures 11–12 ).

**Intraspecific variation.** Body measurements vary in total length from 2.16 to 2.25 mm; maximum width 1.38–1.47 mm; elytral length 1.44–1.59 mm; maximum width of pronotum 1.14–1.20 mm.

**Type-material.** Holotype: male (DZRJ Coleoptera 2967) (22°19'20.27''S 44°40'12.15''W), “Brasil, MG, Itamonte, Serra Negra, alameda das bromélias, 3.xi.2007, Ferreira-Jr N. leg.” / “Holotipo” [plastic red label] / “2967” [plastic red label]; Paratypes: 8 exs (DZRJ Coleoptera 2968) (22°19'20.27''S 44°40'12.15''W), “Brasil, MG, Itamonte, Serra Negra, bromé- lia, 9.iv.2005, Ferreira-Jr. N. & Santos A.D.” / “Paratipo” [plastic red label] / “2968” [plastic black label]; 8 exs. (DZRJ Coleoptera 2969) (22°19'20.27''S 44°40'12.15''W), “Brasil, MG, Maciço de Itatiaia, alameda das bromélias, 13.xi.2008, Bromélia, Ferreira-Jr N. & Clarkson B.” / “Paratipo” [plastic red label] / “2969” [plastic black label]; 10 exs. (MZSP) (22°19'35.09''S 44°39'59.28''W), “Brasil, RJ, Itamonte, Bromélia, 3.xi.2007, Ferreira-Jr N.” / “Paratipo” [plastic red label]; 1 ex. (DZRJ Coleoptera 2627) (22°58'16.70"S 43°1'53.83"W), “RJ; Niterói, Itaquatiara, em bromélia, 17.ix.2000, Luz. A.” / “Paratipo” [plastic red label] / “2627” [plastic blue label].

**Etymology.** The specific epithet is the Latin adjective **grandis,** meaning “big” or “large,” and the Latin adjective **niger** , referring to the species *Desmopachria nigra* Zimmermann, 1923, which is very similar in black color but smaller in size.

**Taxonomic notes.** This new species belongs to the *D. portmanni* group **sensu**Miller (2001) in having the prosternal process sexually dimorphic, male process apically strongly forked, area between rami forming a deep pit, female process not as in male, and the characteristic genitalia with paramere curved, with a row of bristles on the internal edge. This is a bromelicolous species and has a black vitreous color characteristic of the two other bromelicolous species, *D. nigra* and *D. laesslei* Young,

1981. However, compared to the types of *D. nigra* and the bibliography of *D. laesslei* , the new species proved to be bigger. *Demo- pachria laesslei* belongs to *D. convexa* group and has a different type of genitalia, with subapical articulable process in lateral lobes, and the type of *D. nigra* has no more genitalia, therefore not allowing comparison between the two species, but the species only reaches 2.00 mm in size.

**Geographic distribution** : So far known only from the Minas Gerais and Rio de Janeiro States in Brazil

*Desmopachria itamontensis* **sp. nov.**

( Figures 13–15 )

**Figures 13–15.**
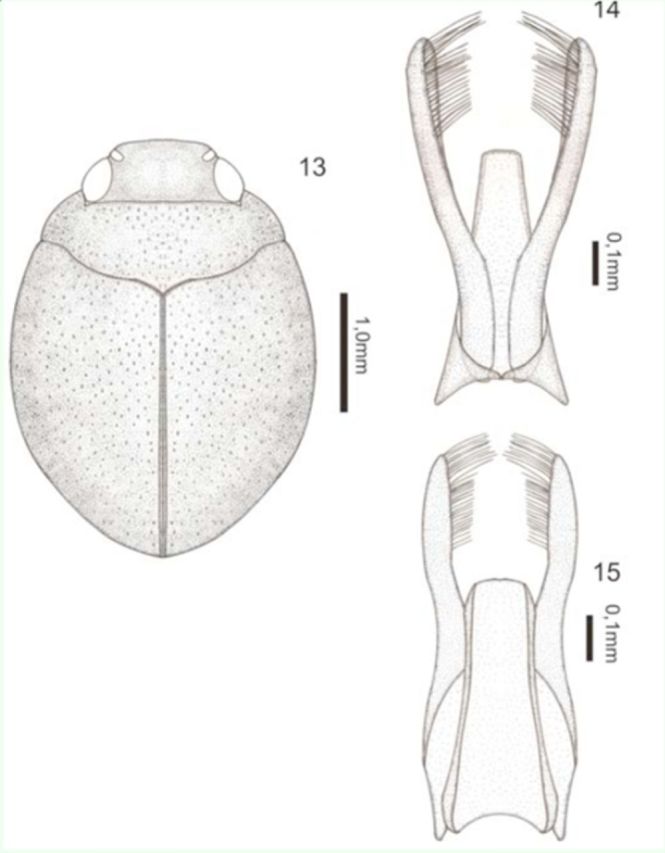
*Desmopachria itamontensis* **sp. nov.** 13) Habitus, dorsal. 14) Male genitalia, ventral view. 15) Male genitalia, dorsal view. High quality figures are available online.

**Description.** Holotype male: total length 2.70 mm; maximum width 1.80 mm; elytral length 1.80 mm; maximum width of the pronotum 1.40 mm. Dorsal and ventral surface dark brown. ( Figure 13 )

Body rounded. Head inconspicuously punctate; lightly truncate and beaded; antennomeres 6–10 slightly expanded in apical half. Pronotum short and wide, with course punctures except for an area in each side of the disc; without basal striae and lateral bead; prosternal process apically strongly forked, area between rami forming a deep pit; metasternum, metacoxae, and ventrites with coarse punctures except for the area around the coxal line. Elytron with course scattered punctures. Aedeagus a little more than half the length of paramere; margined with base wide and narrowing to apex; apex truncated. Paramere thin, distal third with inner edge flattened and with a dense row of setae ( Figures 14–15 ).

**Intraspecific variation** . Total length 2.40– 2.70 mm; maximum width 1.60–1.80 mm; elytral length 1.60–1.90 mm; maximum width of the pronotum 1.20–1.40 mm.

**Type material.** Holotype: male (DZRJ Coleoptera 3164) (22°20'37.05"S 44°41'33.04"W), “Brasil, MG; Itamonte, crenon-couves, 08.ix.2000, Ferreira-Jr N. & Nicolini L.B.” / “Holotipo” [plastic red label] / “3164” [plastic red label]; Paratypes: 15 exs. (DZRJ Coleoptera 3165) (22°20'37.05"S 44°41'33.04"W), “Brasil,MG, Itamonte, crenon-couves, 18.x.1997, Ferreira-Jr N” / “Paratipo” [plastic red label] / “3165” [plastic black label]; 2 exs. (DZRJ Coleoptera 3166) (22°20'37.05"S 44°41'33.04"W), “Brasil, MG, Itamonte, crenon-couves II, 22.ix.2003, Ferreira-Jr N”/ “Paratipo” [plastic red label] / “3166” [plastic black label]; 12 exs. (DZRJ Coleoptera 3167) (22°20'59.11"S 44°41'55.13"W), “Itamonte-MG, 13.ix.96, Equipe Entomologica leg, (Córrego abaixo da cabana, Rio Aiuruoca)” / “Paratipo” [plastic red label] / “3167” [plastic black label]; 2 exs. (DZRJ Coleoptera 3168) (22°20'37.05"S 44°41'33.04"W), “Brasil, MG-Itamonte, crenon couves, 08.ix.2000, Caramaschi F.P.” / “Paratipo” [plastic red label] / “3168” [plastic black label]; 15 exs. (Coleoptera 3169) (22°20'37.05"S 44°41'33.04"W), “Brasil, MG, Itamonte, crenon-couves, 08.x.2000, Ferreira-Jr N. & Nicolini L.B.” / “Paratipo” [plastic red label] / “3169” [plastic black label]; 6 exs. (DZRJ Coleoptera 3170) (22°20'37.05"S 44°41'33.04"W) “Brasil, Minas Gerais, Itamonte, c.couves, depois do acampamento, 12.ix.1998, Entomologia leg., água parada” / “Paratipo” [plastic red label] / “3170” [plastic black label]; 2 exs. (DZRJ Coleoptera 3171) (22°20'37.05"S 44°41'33.04"W), “Brasil, MG, Itamonte, tributário do rio Aiuruoca, crenon-couves, 13.x.2001, Ferreira-Jr N.” / “Paratipo” [plastic red label] / “3171” [plastic black label].

**Etymology.** The specific epithet **itamontensis** is the gentilic to natives from Itamonte municipality, the place of origin of the type material.

**Taxonomic notes.** This new species belongs to the *D. portmanni* group **sensu**Miller (2001) in having the prosternal process sexually dimorphic, male process apically strongly forked, area between rami forming a deep pit, female process not as in male, and the characteristic genitalia, with paramere curved and with a row of bristles. This species is very similar in the shape of the body and the genitalia to *D. grammosticta*** sp. nov.** , but the pronotum has a pair of areas without punctures in each side of disc, and the punctures of elytra do not form rows. The genitalia are almost identical, except that the aedeagus reaches a little over half of parameres, while in *D. grammosticta*** sp. nov.** it reaches halfway; *D. itamontensis*** sp. nov.** has the most dense rows of setae.

**Geographic distribution** : So far known only from the Minas Gerais State in Brazil

*Desmopachria leptophallica* **sp. nov**

( Figures 16–18 )

**Figures 16–18.**
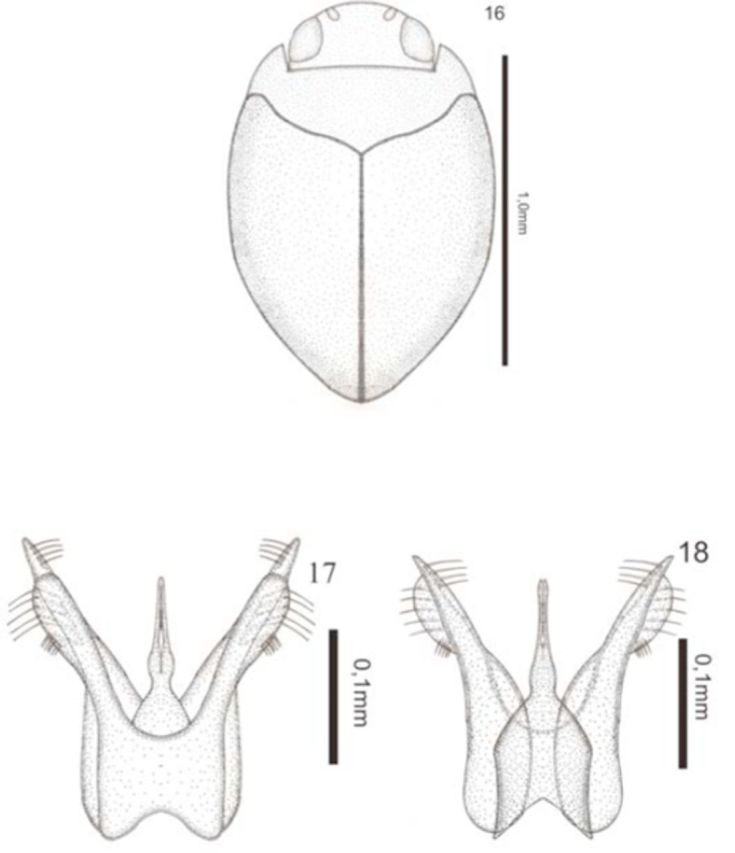
*Desmopachria leptophallica* **sp. nov.** 16) Habitus, dorsal. 17) Male genitalia, ventral view. 18) Male genitalia, dorsal view. High quality figures are available online.

**Description** *.* Holotype male: total length 1.03 mm; maximum width 0.70 mm; elytral length 0.65 mm; maximum width of the pronotum 0.55 mm. Head yellowish-brown, in dorsal view. Pronotum and elytra lightbrown, elytra with dark sutures ( Figure 16 ). Ventrally yellowish.

Body rounded. Head fine, shallow, and very sparsely punctuate; clypeus with very fine margin, slightly truncated; antennomeres 5– slightly expanded in apical half. Pronotum short and wide, shallow and densely punctuate, without basal striae and lateral bead. Metasternum and metacoxae with fine, shallow, and very sparse punctures. Elytron coarse, shallow, and densely punctuate. Ventrites finely and sparsely punctuate; last ventrite transversely impressed. Aedeagus with basal half broad and apex half strict, narrowing abruptly in the middle followed by a slight swelling, apex with a long incision in the center get up close to the swelling. Paramere with the apical half divided two lobes; dorsal lobe straight, with few long setae on inner margin near the apex; ventral lobe rounded, with few setae and a row of short setae on the outer margin ( Figures 17–18 ).

**Intraspecific variation** . Body measurements can vary in total length from 1.03 to 1.06 mm; maximum width 0.64–0.74 mm; elytral length 0.65–0.68 mm; maximum width of the pronotum 0.54–0.56 mm.

**Type-material** . Holotype: male (DZRJ Coleoptera 2232) (22°55'08.73"S 42°49'05.83"W), “Brasil, RJ; Restinga de Maricá, Maricá 3.i.1992, Ferreira-Jr N.” / “Holotipo” [plastic red label] / “2232” [plastic red label]; Paratypes: 3 exs. (DZRJ Coleoptera 119) (22°55'08.73"S 42°49'05.83"W), “Restinga de Maricá, Maricá, 8.ix.1988, Ferreira-Jr N.” / “Paratipo” [plastic red label] / “119” [plastic black label]; 1 ex. (DZRJ Coleoptera 120) (22°55'08.73"S 42°49'05.83"W), “Restinga de Maricá, Maricá, 8.ix.1988, N. Ferreira-Jr” / “Paratipo"[plastic red label] / "120" [plastic black label]; 4 exs. (DZRJ Coleoptera 121) (22°55'08.73"S 42°49'05.83"W), “Restinga de Maricá, Maricá, 15.x.1988, Ferreira-Jr N.” / “Paratipo” [plastic red label] / “121” [plastic black label]; 1 ex. (DZRJ Coleoptera 122) (22°55'08.73"S 42°49'05.83"W), “Restinga de Maricá, Maricá, 2.vi.1989, Ferreira-Jr N.” / “Paratipo” [plastic red label] / “122” [plastic black label]; 1 ex. (DZRJ Coleoptera 123) (22°55'08.73"S 42°49'05.83"W), “Restinga de Maricá, Maricá, RJ, 19.vii.1988, Ferreira-Jr N.” / “Paratipo” [plastic red label] / “123” [plastic black label]; 15 exs. (DZRJ Coleoptera 124) (22°55'08.73"S 42°49'05.83"W), “Restinga de Maricá, Maricá, 23.ix.198, Ferreira-Jr N.” / “Paratipo” [plastic red label] / “124” [plastic black label]; 2 exs. (DZRJ Coleoptera 125) (22°55'08.73"S 42°49'05.83"W), “Restinga de Maricá, Maricá, RJ, 27.i.1990, Ferreira-Jr N.” / “Paratipo” [plastic red label] / “125” [plastic black label]; 1 ex. (DZRJ Coleoptera 126) (22°55'08.73"S 42°49'05.83"W), “Restinga de Maricá, Maricá, RJ, 4.iv.1990, Ferreira-Jr N.” / “Paratipo” [plastic red label] / “126” [plastic black label]; 5 exs. (DZRJ Coleoptera 328) (22°55'08.73"S 42°49'05.83"W), “Restinga de Maricá, Maricá - RJ, 5.ix.1990, FerreiraJr N. leg.” / “Paratipo” [plastic red label] / “328” [plastic black label]; 1 ex. (DZRJ Coleoptera 338) (22°55'08.73"S 42°49'05.83"W), “Restinga de Maricá, Maricá - RJ, 17.xii.1986, Ferreira-Jr N. & da Silva E.R.” / “Paratipo” [plastic red label] / “338” [plastic black label]; 1 ex. (DZRJ Coleoptera 521) (22°54'45.21''S 43°42'40.55''W), “Canal do Itá, Santa Cruz, Rio de Janeiro - RJ, 29.IX.1989; B. Golt- new” / “Paratipo” [plastic red label] / “521” [plastic black label]; 24 exs. (DZRJ Coleoptera 567) (22°55'08.73"S 42°49'05.83"W), “Restinga de Maricá, Maricá - RJ; 15.XI.1991, Ferreira-Jr N.” / “Paratipo” [plastic red label] / “567” [plastic black label]; 15 exs. (DZRJ Coleoptera 590) (22°55'08.73"S 42°49'05.83"W), “Restinga de Maricá, Maricá - RJ, 3.i.1992, Ferreira-Jr N.” / “Paratipo” [plastic red label] / “590” [plastic black label]; 6 exs. (DZRJ Coleoptera 592) (22°55'08.73"S 42°49'05.83"W), “Restinga de Maricá, Maricá - RJ, 3.i.1992, Ferreira-Jr N.” / “Paratipo” [plastic red label] / “592” [plastic black label]; 5 exs. (DZRJ Coleoptera 599) (22°55'08.73"S 42°49'05.83"W), “Restinga de Maricá, Maricá - RJ, 3.i.1992, Ferreira-Jr N / “Paratipo” [plastic red label] / “599” [plastic black label]; 3 exs. (DZRJ Coleoptera 2697) (22°55'08.73"S 42°49'05.83"W), “Maricá - RJ, 21.viii.1996, poça 3, Ferreira-Jr N.” / “Paratipo” [plastic red label] / “2697” [plastic blue label]; 1 ex. (DZRJ Coleoptera 2698) (22°55'08.73"S 42°49'05.83"W), “Maricá - RJ, 21.viii.1996, poça 3, FerreiraJr N.” / “Paratipo” [plastic red label] / “2698” [plastic blue label]; 2 exs. (DZRJ Coleoptera 2699) (22°55'08.73"S 42°49'05.83"W), “Maricá - RJ,10.xi.1995,poça aeronáutica, Ferreira-Jr N.” / “Paratipo” [plastic red label] / “2699” [plastic blue label]; 2 exs. (DZRJ Coleoptera 2701) (22°55'08.73"S 42°49'05.83"W), “Maricá - RJ, 21.viii.1996, poça (aeronáutica), Ferreira-Jr N.” / “Paratipo” [plastic red label] / “2701” [plastic blue label]; 1 ex. (DZRJ Coleoptera 2702) (22°55'08.73"S 42°49'05.83"W), “Maricá, 21.viii.1996, poça 3, Ferreira-Jr N.” / “Paratipo” [plastic red label] / “2702” [plastic blue label].

**Etymology** . The specific epithet is the Greek adjective **leptos,** meaning “thin,” and the Greek noun **phallos** , meaning “aedeagus,” referring to thin apex of the aedeagus.

**Taxonomic notes.** This species belongs to the *Desmopachria nitida* group **sensu**Miller (2001) because of the lateral lobes deeply bifid with apex divided into two long rami. This new species is similar to *D. margarita* Young and *D. psarammo*Miller, 1999 , however, it differs from *D. margarita* by the lack of rounded area in aedeagus and the general shape of paramere in position of bristles in lobes, and from *D. psarammo* by the absence of pattern of maculae in the elytra, the smaller size, and the general shape of aedeagus.

**Geographic distribution** : So far known only from the Rio de Janeiro State in Brazil

*Desmopachria stethothrix* **sp. nov.**

( Figures 19–25 )

**Figures 19–25.**
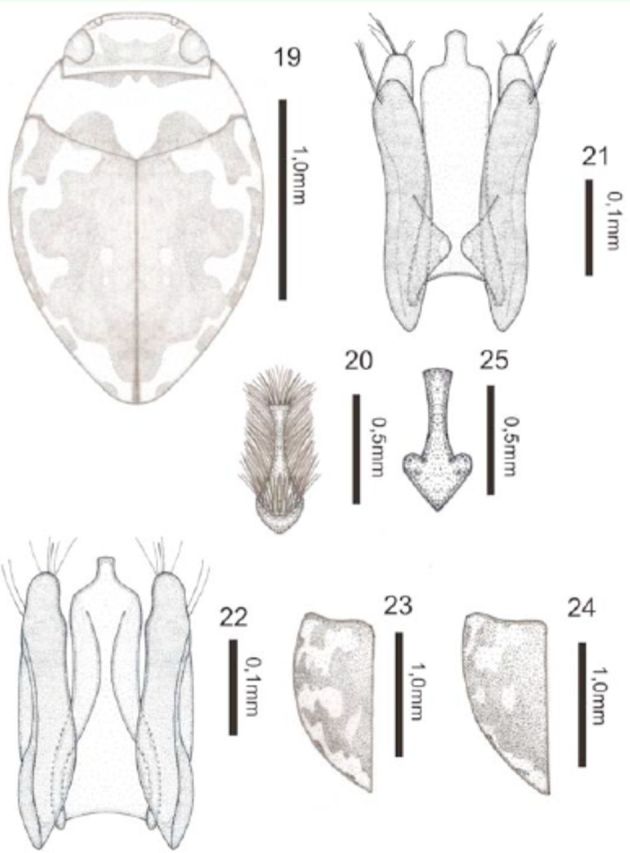
*Desmopachria stethothrix* **sp. nov.** 19) Habitus, dorsal. 20) Male prosternal process. 21) Male genitalia, ventral view. 22) Male genitalia, dorsal view. 23–24) Variations in pattern of spots on elytra. 25) Female prosternal process. High quality figures are available online.

**Description.** Holotype male: total length 2.28 mm; maximum width 1.50 mm; elytral length 1.38 mm; maximum width of the pronotum 1.05 mm. Head yellow. Pronotum yellow with pair of basal dark brown spots. Elytron yellow with brown maculae; a large, irregular brown macula on the disc reaching the base with four expansions along the length, a brown subhumeral macula stretched longitudinally from the base, four brown maculae along the lateral, the first, third, and fourth short stretch along the side ( Figure 19 ). Ventral surface yellow.

Body rounded. Head inconspicuously punctate; clypeus not truncate and beaded; antennomeres 7–9 slightly expanded in apical half. Pronotum short and wide, very fine and inconspicuously punctate, basal striae long and undulated, and lateral beads absent; prosternal process setiform, skirted by a row of long setae ( Figure 20 ); metasternum, metacoxae, and abdomen with scattered fine punctures. Elytron with very fine and obscure punctures. Aedeagus with basal half wide and apical half lined, apex truncate with a straight truncated apical expansion. Paramere wide with undulating edges, in ventral view apex bilobated with rows of setae, in dorsal view only the largest lobe is visible ( Figures 21–22 ).

**Intraspecific variation** . The elytra pattern of colors can vary in the extent depending on the melanism in each specimen ( Figures 23–24 ). The females do not have the rows of bristles in prosternal process ( Figure 25 ). Body measurements variable in male, the total length 2.23–2.38 mm; maximum width 1.50–1.55 mm; elytral length 1.45–1.50 mm; maximum width of pronotum 1.30–1.33 mm and in female the total length 2.05–2.15 mm; maximum width 1.38–1.50 mm; elytral length 1.25–1.50 mm; maximum width of pronotum 1.13–1.25 mm.

**Type-material** Holotype: male (INPA) (00°48'2.77''N 63°29'1.32''W), “Brasil, AM, Barcelos, Comunidade Ukuki, sítio do Sr. Miranda, Rio Jauapéri, 00,80057oN 63,49009oW, 22.vii.2009, Ferreira-Jr N.” / “Holotipo” [plastic red label]; Paratypes: 41 exs. (INPA) (00°48'2.77''N 63°29'1.32''W), “Brasil, AM, Barcelos, Comunidade Ukuki, sítio do Sr. Miranda, Rio Jauapéri, 22.vii.2009, Ferreira-Jr N.” / “Paratipo” [plastic red label]; 30 exs. (DZRJ Coleoptera 2954) (00°48'02.77''N 63°29'01.32''W), “AM, Barcelos, Comunidade Ukuki, sítio do Sr. Miranda, Rio Jauaperi, 22.vii.2009, Ferreira-Jr N.” / “Paratipo” [plastic red label] */* “2954” [plastic black label]; 18 exs. (DZRJ Coleoptera 2955) (00°48'02.77''N 63°29'01.32''W), “BR, AM, Barcelos, Comunidade Ukuki, sítio do Sr. Miranda, Rio Ukuki, 23.vii.2009, Kinom, Ferreira-Jr N.” / “Paratipo” [plastic red label] */* “2955” [plastic black label].

**Etymology.** The specific epithet is the Greek noun **stethos,** meaning “chest,” and the Greek adjective **thrix** , meaning “hairy” or “shaggy,” referring to the prominent pubescence on the prosternal process surface of male.

**Taxonomic notes.** This new species belongs to the *D. glabricula* group **sensu**Miller (2001) in having median and lateral lobes very strongly robust and heavily sclerotized. The only species with colored elytra in this group is *D. volvata* Young, 1981, but the pattern of maculae do not have all characteristic expansions of *D. stethothrix***sp. nov***.* The prosternal process and genitalia in this new species are unique and distinctive.

**Geographic distribution** : So far known only from the Amazonas State in Brazil

*Desmopachria ukuki* **sp. nov.**

( Figures 26–28 )

**Figures 26–28.**
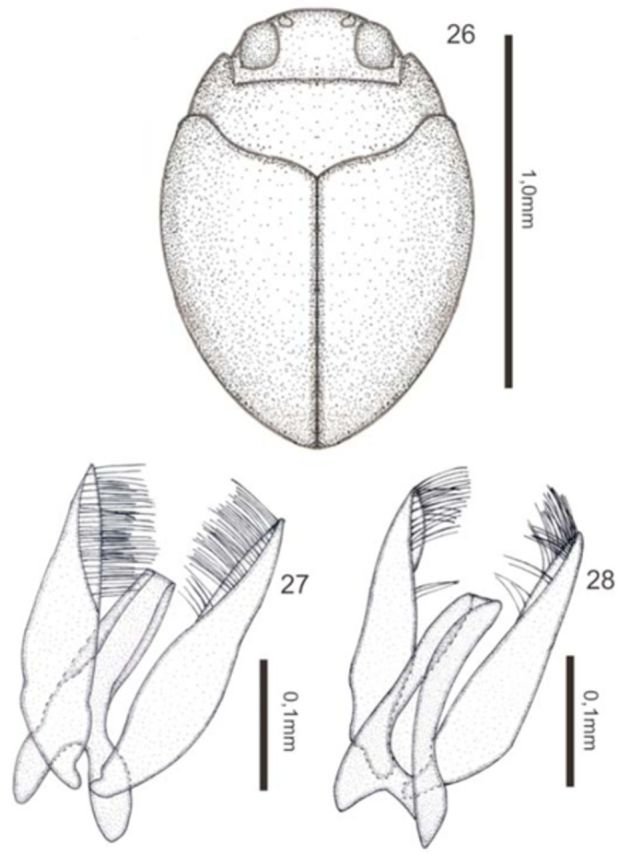
*Desmopachria ukuki* **sp. nov.** 26) Habitus, dorsal. 27) Male genitalia, ventral view. 28) Male genitalia, dorsal view. High quality figures are available online.

**Description.** Holotype male: total length 2.03 mm; maximum width 1.50 mm; elytral length 1.38 mm; maximum width of the pronotum 1.05 mm. Head and pronotum light brown. Elytron light brown with dark maculae for transparency ( Figure 26 ). Ventral surface light brown.

Body rounded. Head inconspicuously punctate; clypeus indistinctly beaded; antennomeres 7–9 slightly expanded in apical half. Pronotum short and wide, very fine and inconspicuously punctate, without basal striae and lateral bead; prosternal process apically strongly forked, area between rami forming a deep pit. Metasternum, metacoxae, and abdomen with scattered fine punctures. Elytron with fine and obscure punctures. Last ventrite with an undulated suture. Aedeagus with broad base, apical half narrowed and margined, apex truncated. Paramere wide almost thin, inner margin with a tuft of setae in distal third. ( Figure 27–28 )

**Intraspecific variation.** Body measurements vary, total length 2.03–2.05 mm; elytral length 1.38–1.50 mm.

**Type material** Holotype: male (INPA) (00°45'25.60''N 63°26'26.02''W), “Brasil, AM, Barcelos, acampamento base, poça com folhiço, (B808) 23.viii.2009, Ferreira-Jr N.” / “Holotipo” [plastic red label]; Paratypes: 2 female (DZRJ Coleoptera 2956) (00°45'25.60''N 63°26'26.02''W), “Brasil, AM, Barcelos, acampamento base, 5.viii.2009, poça com folhiço, (B808), Ferreira-Jr N.” / “Paratipo” [plastic red label] / “2956” [plastic black label].

**Etymology.** The specific epithet is an apposition noun that refers to the Ukuki community, the place of origin of the type material.

**Taxonomic notes.** This new species belongs to the *D. portmanni* group **sensu**Miller (2001) in having the prosternal process sexually dimorphic, male process apically strongly forked, area between rami forming a deep pit, female process not as in male, and the characteristic genitalia, with paramere curved and with a row of bristles on the internal edge. This species is only comparable with *D. speculum*Sharp, 1887 , which has a shape of body and genitals very similar, but can be separated by the large body size of *D. ukuki***sp. nov***.*

**Geographic distribution** : So far known only from the Amazonas State in Brazil

New records from Brazil

*Desmopachria aldessa* **Young, 1980**

**PARÁ STATE: Flona Carajás; Parauapebas municipality;** 4 exs. (DZRJ Coleoptera 2040) (06°06'08.37''S 50°11'06.97''W), Serra Norte; N4A, 09.ix.2006, Dumas L.L. & Ferreira-Jr N. leg.; 2 exs. (DZRJ Coleoptera 2045) (06°5'43.00''S 50°11'29.25''W), N4D, 21.ix.2007, Alecrim V.P. & Ferreira-Jr N. leg.; 1 ex. (DZRJ Coleoptera 2042) (06°04'57.10''S 50°08'05.25''W) Buritizal 1, 23.iii.2003, Ferreira-Jr N. leg.; 3 exs. (DZRJ Coleoptera 2043) (06°04'57.10''S 50°08'05.25''W) Buritizal 1, 16.ix.2006, Dumas L.L. & Ferreira-Jr N. leg *.* ; 4 exs. (DZRJ Coleoptera 2044) (06°04'57.10''S 50°08'05.25''W) Buritizal 1, 25.ix.2007, Alecrim V.P. & Ferreira-Jr N. leg *.* ; 33 exs. (DZRJ Coleoptera 2047) (06°04'57.10''S 50°08'05.25''W) Buritizal 1, 29.ix.2007, Alecrim V.P. & Ferreira-Jr N. leg.; 8 exs. (DZRJ Coleoptera 2046) Buritizal 2 (06°04'13.39''S 49°56'59.61''W), poça com folhiço, 08.ix.2006, Dumas L.L. & Ferreira-Jr N. leg.; 2 exs. (DZRJ Coleoptera 2048) Buritizal 2 (06°04'13.39''S 49°56'59.61''W), 24.ix.2007, Alecrim V.P. & Ferreira-Jr N. leg. **Canaã dos Carajás municipality: Serra Sul;** , 1 ex. (DZRJ Coleoptera 2041) entre s11ca e s11cb (06°22'08.45''S 50°23'10.86''W), 15.ix.2006, Ferreira-Jr N. leg.

*Desmopachria amyae* **Miller, 2001**

**PARÁ STATE: Flona Carajás; Parauapebas municipality; Serra Norte;** 1 ex. (DZRJ Coleoptera 2050) (06°04'13.39''S 49°56'59.61''W), lago gelado, 25.ix.2007, Alecrim V.P. & Ferreira-Jr N. leg. **Canaã dos Carajás municipality; Serra Sul;** 1 ex. (DZRJ Coleoptera 2035) (06°04'13.39''S 49°56'59.61''W), Córrego leito de pedra entre s11a e s11b, 27.ix.2007, Alecrim V.P. & Ferreira-Jr N. leg.; 11 exs. (DZRJ Coleoptera 2036) (06°04'13.39''S 49°56'59.61''W), Córrego leito de pedra entre s11a e s11b, 27.ix.2007, Alecrim V.P. & Ferreira-Jr N.leg.; 20 exs. (DZRJ Coleoptera 2037) (06°04'13.39''S 49°56'59.61''W), córrego leito de pedra entre s11a e s11b, 15.ix.2006, Dumas L.L. & Ferreira-Jr N. leg.; 5 exs. (DZRJ Coleoptera 2038) (06°04'13.39''S 49°56'59.61''W), córrego leito de pedra entres11a e s11b, 15.ix.2006, Dumas L.L. & Ferreira-Jr N. leg. **Curionópolis municipality; Serra Pelada;** 1 ex. (DZRJ Coleoptera2039) (06°04'13.39''S 49°56'59.61''W), LA, 20.ix.2007, Alecrim V.P. & Ferreira-Jr N. leg.

*Desmopachria chei* **Miller, 1999**

**MINAS GERAIS STATE: Nova Viçosa municipality;** 7 exs. (DZRJ Coleoptera 2705) (20°45'10.45''S 42°53'06.48''W), UFV, Reservatório de segmentação agro, 14.vii.1997, Carvalho A.L. & Ferreira Jr N. leg.; 1 ex. (DZRJ Coleoptera 2706), (20°45'10.45''S 42°53'06.48''W), UFV, Reservatório de segmentação agro, 14.vii.1997, Carvalho A.L. & Ferreira-Jr N. leg. **RIO DE JANEIRO STATE: Maricá municipality;** 1 ex. (DZRJ Coleoptera 112) (22°57'19.48''S 42°16'06.49''W), Restinga de Maricá, 07.vi.1988, Ferreira-Jr N. leg.; 1 ex., (DZRJ Coleoptera 113) (22°57'19.48''S 42°16'06.49''W), Restinga de Maricá,, 03.ix.1988, Ferreira-Jr N. leg.; 33 exs. (DZRJ Coleoptera 115) (22°57'19.48''S 42°16'06.49''W), Restinga de Maricá,04.iv.1990, Ferreira-Jr N. leg.; 3 exs. (DZRJ Coleoptera 327) (22°57'19.48''S 42°16'06.49''W), Restinga de Maricá, 05.ix.1990, Ferreira-Jr N. leg.; 6 exs. (DZRJ Coleoptera 457) (22°57'19.48''S 42°16'06.49''W), Restinga de Maricá,, 18.viii.1991, Ferreira-Jr N. leg.; 2 exs. (DZRJ Coleoptera 569) (22°57'19.48''S 42°16'06.49''W), Restinga de Maricá, 15.xi.1991, Ferreira-Jr N. leg. **Teresópolis municipality;** 6 exs. (DZRJ Coleoptera 600) (22°26'48.62''S 42°53'21.87''W), Serra do Subaio, 7.vi.1996, Ferreira Jr. N. Leg.; 17 exs. (DZRJ Coleoptera 2707) (22°20'25.47''S 42°33'26.52''W), Faz. Vale da Revolta, 12.x.1996, N.Ferreira-Jr leg.

*Desmopachria fossulata* **Zimmermann,** **1928**

**RIO DE JANEIRO STATE: Teresópolis municipality;** 2 exs. (DZRJ Coleoptera 353) (22°24'42.78''S 42°58'02.56''W), Faz. Vale da Revolta, 11.i.1990, Ferreira-Jr N. leg.; 2 exs. (DZRJ Coleoptera 2704) (22°24'42.78''S 42°58'02.56''W), Faz. Vale da Revolta, 12.x.1996, Carvalho A.L. & Ferreira-Jr N. leg.

*Desmopachria granoides* **Young, 1986**

**RIO DE JANEIRO STATE: Teresópolis municipality;** 1 ex. (DZRJ Coleoptera 2693) (22°24'42.78''S 42°58'02.56''W), poça no caminho do hotel, 30.iii.1996, Ferreira-Jr N **.** leg **. SÃO PAULO STATE: Ubatuba municipality;** 21 exs. (DZRJ Coleoptera 2671), (23°21'33.70''S 44°50'30.52''W), Parque Estadual da Serra do Mar, Núcleo Picinguaba, 04.x.2006, Braga R.B. & Ferreira-Jr N. leg.; 17 exs. (DZRJ Coleoptera 2695) (23°21'33.70''S 44°50'30.52''W), Parque Estadual da Serra do Mar, Núcleo Picinguaba, 17.vi. 2004, Braga R.B. leg

*Desmopachria laevis* **Sharp, 1882**

**MINAS GERAIS STATE: P.N. do Caparaó; Alto Caparaó;** 45 exs. (DZRJ Coleoptera 3172) (20°23'17.30''S 41°51'13.10''W), Alojamento portaria, 05.x.2010, Ferreira-Jr N. leg. **RIO DE JANEIRO STATE: Maricá municipality;** 3 exs. (DZRJ Coleoptera 119) (22°57'19.48''S 42°16'06.49''W), Restinga de Maricá, 8.ix.1988, Ferreira-Jr N. leg.; **Rio de Janeiro city** , **Parque Nacional da Tijuca** , 18 exs. (DZRJ Coleoptera 384) (22°57'47.28''S 43°14'40.52''W), 10.i.1991, Ferreira-Jr N. leg.; 1 ex. (DZRJ Coleoptera 2684) (22°57'47.28''S 43°14'40.52''W),19.ii.1992, Ferreira-Jr N. leg.; **PARNASO; Teresópolis municipality;** 14 exs. (DZRJ Coleoptera 3173) (22°26'53.11S 42°59'04.77''W) poças marginais ao rio Paquequer, 12.v.2008, Ferreira-Jr N. leg. **RIO GRANDE DO SUL STATE: Santiago municipality;** 7 exs. (DZRJ Coleoptera 3174) (29°35'98.76''S 54°73'0.40''W), Ernesto Alves, 30.iv.2011, Barbosa J.F. leg.

*Desmopachria margarita* **Young, 1990**

**PARÁ STATE: FLONA Carajás; Parauapebas municipality; Serra Norte;** 11 exs. (DZRJ Coleoptera 2052) (06°06'07.92''S 50°08'20.89''W), N4A, 4.iii.2008, Ferreira-Jr N. leg.; 40 exs. (DZRJ Coleoptera 2061) (06°06'07.92''S 50°08'20.89''W), N4A, 25.ix.2007, Alecrim V.P. & Ferreira-Jr N. leg.; 2 exs. (DZRJ Coleoptera 2070) (06°06'07.92''S 50°08'20.89''W), N4A, 9.ix.2006, Dumas L.L. & Ferreira-Jr N. leg.; 2 exs. (DZRJ Coleoptera 2508) (06°06'07.92''S 50°08'20.89''W), N4A, 24.ix.2007, Alecrim V.P. & Ferreira-Jr N. leg.; 4 exs. (DZRJ Coleoptera 2511) (06°06'07.92''S 50°08'20.89''W), N4A, 24.iii.2006, Ferreira-Jr N. leg.; 3 exs. (DZRJ Coleoptera 2515) (06°06'07.92''S 50°08'20.89''W), N4A, 9.ix.2006, Ferreira-Jr N. leg.; N4B, (06°06'7.57"S 50°11'19.17"W), 2 exs. (DZRJ Coleoptera 2077) (06°06'07.92''S 50°08'20.89''W), N4A, x.2005, Ferreira-Jr N. leg.; ,4 exs. (DZRJ Coleoptera 2051) (06°05'43.00''S 50°11'29.25''W), N4D, 27.ii.2008, Santos A. & Ferreira-Jr N. leg.; 19 exs. (DZRJ Coleoptera 2068) (06°06'07.92''S 50°08'20.89''W), N4A, 21.ix.2007, Alecrim V.P. & Ferreira-Jr N. leg.; 7 exs. (DZRJ Coleoptera 2069) (06°06'07.92''S 50°08'20.89''W), N4A, 21.ix.2007, Alecrim V.P. & Ferreira-Jr N. leg.; 2 exs. (DZRJ Coleoptera 2072) (06°06'07.92''S 50°08'20.89''W), N4A, 24.ii.2006, Ferreira-Jr N. leg.; 17 exs. (DZRJ Coleoptera 2079) (06°06'07.92''S 50°08'20.89''W), N4A, 22.iii.2007, Alecrim V.P. & Ferreira-Jr N. leg.; 1 ex. (DZRJ Coleoptera 2509) (06°06'07.92''S 50°08'20.89''W), N4A, 21.ix.2007, Alecrim V.P. & Ferreira-Jr N. leg.; 45 exs. (DZRJ Coleoptera 2518) (06°06'07.92''S 50°08'20.89''W), N4A, 27.ii.2008, Alecrim V.P. & Ferreira-Jr N. leg.; 38 exs. (DZRJ Coleoptera 2519) (06°06'07.92''S 50°08'20.89''W), N4A, 24.iii.2006, Ferreira-Jr N. leg.; 1 ex. (DZRJ Coleoptera 2058) (06°06'12.30''S 50°11'15.64''W) N4E, x.2005, Limnologia UFRJ leg.; 1 ex. (DZRJ Coleoptera 2057) (05°57'56.60''S °12'59.96''W), Lago Gelado, Cela 7, 22.ix.2007, Alecrim V.P. & Ferreira-Jr. N. leg.; 37 exs. (DZRJ Coleoptera 2075) (06°06'07.92''S 50°08'20.89''W), N4A, 28.ix.2007, Dumas L.L. & Ferreira-Jr N. leg.; 10 exs. (DZRJ Coleoptera 2074) (06°05'50.08''S 50°07'50.07''W), N5sul, 22.ii.2006, Ferreira-Jr N. leg.; 1 ex. (DZRJ Coleoptera 2516) (06°05'50.08''S 50°07'50.07''W), N5sul, 22.iii.2006, Ferreira-Jr N. leg.; 24 exs. (DZRJ Coleoptera 2520) (06°05'50.08''S 50°07'50.07''W), N5sul, 22.iii.2006, Ferreira-Jr N. leg.; 1 ex. (DZRJ Coleoptera 2514) (50°12'59.96''W 05°57'56.60''S), lago Gelado, cela 7, 22.ix.2007, Alecrim V.P. & Ferreira-Jr N. leg.; 6 exs. (DZRJ Coleoptera 2078) (05°57'56.60''S 50°12'59.96''W), lago Gelado, caminho para cela 7, 3.iii.2008, Santos A. & Ferreira-Jr N. leg.; , 1 ex. (DZRJ Coleoptera 2053) (06°04'57.10''S 50°08'05.25''W), Buritizal 1, 26.ix.2007, Alecrim V.P. & Ferreira-Jr. N. leg.; 2 exs. (DZRJ Coleoptera 2054) (06°04'57.10''S 50°08'05.25''W), Buritizal 1, 16.ix.2006, Dumas L.L. & Ferreira-Jr. N. leg.; 1 ex. (DZRJ Coleoptera 2059) (06°04'57.10''S 50°08'05.25''W), Buritizal 1, 5.ii.2008, Santos A. & Ferreira-Jr N. leg.; 5 exs. (DZRJ Coleoptera 2060) (06°04'57.10''S 50°08'05.25''W), Buritizal 1, 27.ii.2008, Santos A. & Ferreira-Jr N. leg.; 3 exs. (DZRJ Coleoptera 2062) (06°04'57.10''S 50°08'05.25''W), Buritizal 1, 23.ix.2007, Alecrim V.P. & Ferreira-Jr N. leg.; 349 exs. (DZRJ Coleoptera 2063) (06°04'57.10''S 50°08'05.25''W), Buritizal 1, 11.ix.2006, Ferreira-Jr N. leg.; 3 exs. (DZRJ Coleoptera 2064) (06°04'57.10''S 50°08'05.25''W), Buritizal 1, 23.ix.2007, Alecrim V.P. & Ferreira-Jr N. leg.; 3 exs. (DZRJ Coleoptera 2065) (06°04'57.10''S 50°08'05.25''W), Buritizal 1, 11.ix.2006, Ferreira-Jr N. leg.; 2 exs. (DZRJ Coleoptera 2066) (06°04'57.10''S 50°08'05.25''W), Buritizal 1, 29.ix.2007, Alecrim V.P. & Ferreira-Jr N. leg.; 15 exs. (DZRJ Coleoptera 2067) (06°04'57.10''S 50°08'05.25''W), Buritizal 1, 25.ix.2007, Alecrim V.P. & Ferreira-Jr N. leg.; 1 ex. (DZRJ Coleoptera 2076) (06°04'57.10''S 50°08'05.25''W), Buritizal 1, 28.ix.2007, Alecrim V.P. & Ferreira-Jr N. leg.; 1 ex. (DZRJ Coleoptera 2513) (06°04'57.10''S 50°08'05.25''W), Buritizal 1, 29.ix.2007, Alecrim V.P. & Ferreira-Jr N. leg.; 35 exs. (DZRJ Coleoptera 2521) (06°04'57.10''S 50°08'05.25''W), Buritizal 1, 16.ix.2006, Dumas L.L. & Ferreira-Jr N. leg.; 3 exs. (DZRJ Coleoptera 2055) (06°04'13.39''S 49°56'59.61''W), Buritizal 2, 8.ix.2006, Dumas L.L. & Ferreira-Jr N. leg.; 2 exs. (DZRJ Coleoptera 2512) (06°04'13.39''S 49°56'59.61''W), Buritizal 2, 8.ix.2006, Ferreira-Jr N. leg.; **Canaã dosCarajás municipality; Serra Sul;** 4 exs. (DZRJ Coleoptera 2056) (06°20'40.14''S 50°24'31.70''W) Córrego Leito de Pedra, 29.ii.2008, Santos A. & Ferreira-Jr N. leg.; 1 ex. (DZRJ Coleoptera 2510) (06°17'38.59''S 50°22'4.84''W), Córrego Leito de pedra, 27.iii.2006, Alecrim V.P. & Ferreira-Jr N. leg.; 1 ex. (DZRJ Coleoptera 2071), iii.2007, Ferreira-Jr N. leg.; 9 exs. (DZRJ Coleoptera 2517) (06°20'56.71''S 50°26'54.44''W), S11AA, x.2005, Ferreira-Jr N. leg.; **Curionópolis municipality; Serra Pelada;** 12 exs. (DZRJ Coleoptera 2073), (06°17'38.59''S 52°22'4.84''W), LA, 20.ix.2007, Alecrim V.P. & Ferreira-Jr N. leg.

*Desmopachria volatidisca* **Miller, 2001**

**MINAS GERAIS STATE: Nova Viçosa municipality** , 1 ex. (DZRJ Coleoptera 2667) (20°45'10.45''S 42°53'06.48''W), Universidade Federal de Minas Gerais, Reservatório de Sedimentação, 19.i.1997, da Silva E.R. & Coelho L.B.N. leg.; **Simão Perreira municipality** , 1 ex. (DZRJ Coleoptera 2670) (21°49'7.27''S 43°23'26.82''W), Rio Paraibuna, Faz. Cabuí, 04.ix.2000, Ferreira-Jr N. leg. **RIO DE JANEIRO STATE: Comendador Levy Gasparian municipality** , 1 ex. (DZRJ Coleoptera 2669) (21°49'07.27''S 43°23'26.82''W), Mont Serrat, Rio Paraibuna,, 18-19.x.2000, Ferreira-Jr N. leg.

**Checklist of** *Desmopachria* **species known from Brazil**

Notes: (1) In 1975, Mato Grosso State was divided into Mato Grosso and Mato Grosso do Sul States. In old articles, like Zimmermann (1921), the area currently occupied by Mato Grosso do Sul state is known as Mato Grosso. (2) The material record from Brazil with the specific location of “Santa Rita” weas collected by the Finnish R. F. Sahlberg in August 1850. Nilsson (2013) mentions “Santa Rita [da Floresta],” now district of Cantagalo Municipality, Rio de Janeiro States. Although in August 1850 Sahlberg explored the district of Cantagalo for three months ( Papavero 1973 ), the villa of Santa Rita da Floresta was only founded in 1876 by Portuguese immigrant Captain Bernardo de Souza and his cousins. Second Papavero (1973) , Sahlberg visited the golden mines in Minas Gerais and must have collected in this state from Chapéu d’Uvas to Diamantina (the ancient Tejuco), and Santa Rita would be in this itinerary. In this case, Santa Rita corresponds to Santa Rita Durão District, Mariana Municipality, Minas Gerais State. (3) The material described by Young from “Santa Isabel, Ilha do Bananal, Goias” is in Tocantins State since 1988, when Goias State was divided.

***D. aldessa*** Young, 1980 . Brazil: (Tré- mouilles 1995: 32), Maranhão ( Nilsson 2001 : 217, 2013: 197, Young 1980 : 313), Mato Grosso ( Young 1980 : 312), Pará (*). Trinidad ( Trémouilles 1995 : 32, Young 1980 : 312).

*D. amyae* Miller, 2001 . Brazil: Pará (*). Bolivia ( Miller 2001 : 224, Nilsson 2001 : 218, 2013: 198).

*D. attenuata* Régimbart, 1895 . Brazil: ( Nilsson 2001 : 218, 2013: 219, Régimbart 1895 : 324, Trémouilles 1995 : 33, Young 1980 : 319).

*D. aurea* **Young, 1980** . Brazil: ( Trémouilles 1995 : 32), Maranhão ( Nilsson 2001 : 217,

2013: 197, Young 1980 : 314), Pará ( Young 1980 : 314). Suriname ( Young 1980 : 314).

***D. balfourbrownei*** **Young, 1990** . Brazil: Amazonas ( Nilsson 2001 : 218, 2013: 199, Young 1990b : 43).

***D. balionata*** **Miller, 2005** . Brazil: Amazonas (Braga & Ferreira-Jr 2010: 40), Pará (Braga & Ferreira-Jr 2010: 40). Peru ( Miller 2005 : 43, Nilsson 2013 : 197).

***D. bifasciata*** **Zimmermann, 1921** . Brazil: ( Trémouilles 1995 : 33, Young 1980 : 320), Mato Grosso do Sul ( Nilsson 2001 : 218, 2013: 199, Young 1990b : 46, Zimmermann 1921 : 192).

***D. cavia*** **Braga & Ferreira-Jr, 2010** . Brazil: Amazonas ( Braga &Ferreira-Jr 2010 : 37, 38), Nilsson 2013 : 196), Pará ( Braga & Ferreira-Jr 2010 : 37).

*D. chei* **Miller, 1999** . Brazil: Minas Gerais (*), Rio de Janeiro (*). Bolivia ( Miller 1999 : 351, Nilsson 2001 : 218, 2013: 198).

***D. concolor*** **Sharp, 1882** . Brazil (Tré- mouilles 1995: 31, Young 1980 : 307). Argentina (Régimbart 1903: 48, Trémouilles 1995 : 31). Paraguay ( Trémouilles 1995 : 31). Uruguay ( Nilsson 2001 : 218, 2013: 198, Ré- gimbart 1903: 48, Sharp 1882a : 340, Trémouilles 1995 : 31).

*D. convexa* **( Aubé, 1838** ). Brazil: (Aubé 1938: 480, Nilsson 2001 : 215, 2013: 19, Trémouilles 1995 : 33), “Santa Rita” ( Sharp 1882a : 342), Mato Grosso do Sul (Zimmermann 1921: 206). USA ( Aubé 1838 : 480, Nilsson 2001 : 215, Young 1981b : 2).

Note: *D. convexa* was described from the United States and Brazil, but according to Young (1981b) , “all South American *Desmopachria* differ in male external genitalia” in reference to *D. convexa* , and the records from the USA are probably a different species.

*D. dicrophallica* **sp. nov.** Brazil: Pará.

*D. disticta* **sp. nov.** Brazil: Amazonas.

***D. draco*** **Miller, 1999 .** Brazil: Amazonas ( Braga & Ferreira-Jr 2010 : 40, 41), Pará ( Braga & Ferreira-Jr 2010 : 40, 41). Bolivia ( Miller 1999 : 355, Nilsson 2001 : 216, 2013: 197).

*D. duodentata* **Braga & Ferreira, 2011 .** Brasil: Amazonas ( Braga & Ferreira 2011 : 128, Nilsson 2013 : 198).

***D. ferrugata*** **Régimbart, 1895 .** Brazil: ( Trémouilles 1995 : 31, Young 1980 : 310), Bahia ( Nilsson 2001 : 218, 2013: 198, Ré- gimbart 1895: 323, Young 1990a : 225).

***D. fossulata*** **Zimmermann, 1928 .** Brazil: ( Nilsson 2001 : 218, 2013: 198, Trémouilles 1995 : 31, Young 1980 : 310, Zimmermman 1928: 171), Rio de Janeiro (*).

*D. goias* **Young, 1995 .** Brazil: Tocantins ( Nilsson 2001 : 217, 2013: 198, Young 1995 : 38, 41).

*D. grammosticta* **sp. nov.** Brazil: Amazonas.

***D. granum*** ( Leconte, 1855 ).Brazil: (Tré- mouilles 1995: 33), Mato Grosso do Sul & ( Zimmermann 1921 : 206). Argentina (Tré- mouilles 1995: 33). USA ( Leconte 1855 , Nilsson 2001 : 215, 2013: 196, Trémouilles 1995 : 33, Young 1981b : 4).

***D. grandinigra*** **sp. nov.** Brazil: Minas Gerais, Rio de Janeiro. ***D. granoides*Young, 1986 .** Brazil: (Tré- mouilles 1995: 33), Bahia ( Young 1986 : 271), Mato Grosso ( Nilsson 2001 : 216,

2013: 197, Young 1986 : 271), Mato Grosso do Sul ( Young 1986 : 271), Rio de Janeiro, São Paulo ( Young 1986 : 271). Bolivia ( Trémouilles 1995 : 33, Young 1986 : 271). Suriname ( Trémouilles 1995 : 33, Young 1986 : 271). Trinidad ( Trémouilles 1995 : 33, Young 1986 : 271). Venezuela ( Trémouilles 1995 : 33, Young 1986 : 271).

***D. grouvellei*** **Régimbart, 1895 .** Brazil: ( Trémouilles 1995 : 31), Mato Grosso do Sul ( Zimmermann 1921 : 206). Argentina (Tré- mouilles 1995: 31, Young 1990a : 225). Mexico ( Nilsson 2001 : 218, 2013: 198, Ré- gimbart 1895: 323). Paraguay (Régimbart 1903: 49, Trémouilles 1995 :31, Young 1990a : 225).

***D. hylobates*** **Young, 1993 .** Brazil: Tocantins ( Nilsson 2001 : 218, 2013: 199, Young 1980 : 312, Young 1993 : 246).

***D. iridis*** Young, 1980 .Brazil: ( Trémouilles 1995 : 32), Maranhão ( Young 1980 : 312), Pará ( Nilsson 2001 : 217, 2013: 198, Young 1980 : 312).

***D. itamontensis*** **sp. nov.** Brazil: Minas Gerais.

***D. laevis*** **Sharp, 1882.** Brazil: ( Trémouilles 1995 : 32, Young 1980 : 311), “Boa-sorta” ( Régimbart 1895 : 323), “Santa Rita” (Nilsson 2001: 217, 2013: 198, Sharp 1882a : 341, Young 1995 : 41), Maranhão ( Young 1995 : 41), Minas Gerais (*), Rio de Janeiro (*), Rio Grande do Sul (*). Mexico ( Régimbart 1895 : 323).

Note: Probably “Boa Sorte”. According to d'Orchimont (1942), this locality is in the State of Rio de Janeiro. It is possible that these records correspond to localities in this state, since there are at least two places with the name of Boa Sorte, in the municipalities of Barra Mansa and Cantagalo.

***D. leptophalla*** **sp. nov.** Brazil: Rio de Janeiro.

***D. liosomata*** **Young, 1986 .** Brazil: (Tré- mouilles 1995: 33), Mato Grosso ( Nilsson 2001 : 216, 2013: 19, Young 1986 : 270), Mato Grosso ( Young 1986 : 270).

***D. manus*** **Braga & Ferreira-Jr, 2010 .** Brazil: Amazonas ( Braga & Ferreira-Jr 2010 : 34, Nilsson 2013 : 19), Pará ( Braga & Ferreira-Jr 2010 : 34).

***D. margarita*** **Young, 1990.** Brazil: Pará (*). Panamá ( Nilsson 2001 : 216, 2013: 19, Sharp 1887 : 752, Young 1990b : 47).

*= D. glabricula* Sharp **sensu**Sharp 1887 : 752 nec Sharp 1882b : 18.

***D. minuta*** **Young, 1980 .** Brazil: (Tré- mouilles 1995: 32), Mato Grosso ( Nilsson 2001 : 218, 2013: 198, Young 1980 : 319).

***D. mutata*** **Sharp, 1882.** Brazil: (Tré- mouilles 1995: 32, Young 1980 : 311), Bahia ( Sharp 1882b : 18, Young 1995 : 33, 41).

= *D. bryanstonii* (Clark) **sensu**Sharp 1882a : 342 **nec**Clark 1862 : 175.

***D. nigra*** **Zimmermann, 1923 .** Brazil: ( Trémouilles 1995 : 32, Young 1980 : 311), São Paulo ( Nilsson 2001 : 217, 2013: 198, Zimmermann 1923 : 33).

*D. nigrocapitata* **Braga & Ferreira-Jr,** **2010** .Brazil: Amazonas ( Braga & Ferreira-Jr 2010 : 39, 40, Nilsson 2013 : 199).

***D. nitida*** **Babington, 1841 .** Brazil: (Tré- mouilles 1995: 33, Young 1980 : 319), “Santa Rita” ( Young 1986 : 269), Mato Grosso do Sul ( Zimmermann 1921 : 206), Rio de Janeiro ( Babington 1841 : 17, Nilsson 2001 : 216, 2013: 197, Sharp 1882a : 342). Argentina ( Trémouilles 1995 : 33).

***D. nitidissima*** **Zimmermann, 1928 .** Brazil: ( Nilsson 2001 : 217, 2013: 198, Trémouilles 1995 : 32, Young 1980 : 311, Young 1995 : 43, Zimmermann 1928 : 171). Argentina ( Trémouilles 1995 : 32).

***D. ovalis*** **Sharp, 1882.** Brazil: ( Young 1980 : 319), “Santa Rita” ( Nilsson 2001 : 218, 2013:199, Sharp 1882a : 340). Argentina ( Trémouilles 1995 :33).

***D. paradoxa*** **Zimmermann, 1923 .** Brazil: ( Trémouilles 1995 : 31, Young 1980 : 310), São Paulo ( Nilsson 2001 : 219, 2013: 199, Zimmermann 1923 : 33).

***D. pulvis*** **Guignot, 1958 .** Brazil: (Tré- mouilles 1995: 33, Young 1980 : 320), “Salobra” probably Mato Grosso do Sul ( Guignot 1958 : 35, Nilsson 2001 : 215, 2013: 19, Young 1990b : 47).

***D. ruginosa*** **Young, 1990.** Brazil: Tocantins ( Young 1990a : 228), Mato Grosso ( Nilsson 2001 : 218, 2013: 198, Young 1990a : 228).

***D. signata*** **Zimmermann, 1921 .** Brazil: ( Trémouilles 1995 : 33, Young 1980 : 320), Goiás ( Young 1990b : 44), Mato Grosso do Sul ( Nilsson 2001 : 215, 2013: 196, Young 1990b : 44, Zimmermann 1921 : 192). Colombia ( Young 1990b : 44). Panama ( Young 1990b : 44).

***D. siolii*** **Young, 1980 .** Brazil: ( Trémouilles 1995 : 32), Amazonas ( Nilsson 2001 : 218, 2013: 198, Young 1980 : 318).

***D. stethothrix*** **sp. nov.** Brazil: Amazonas.

*D. striola* **Sharp, 1887** *.* Brazil: ( Young 1990a : 227), Amazonas ( Braga & Ferreira-Jr 2010 : 40, 41), Pará ( Braga & Ferreira-Jr 2010 : 40, 41). Argentina ( Young 1990a : 227). Bolivia ( Young 1990a : 227). Colombia ( Young 1990a : 227). Costa Rica ( Young 1990a : 227). Ecuador ( Young 1990a : 227). Guatemala ( Young 1990a : 227). Panama ( Nilsson 2001 : 218, 2013: 198, Sharp 1887 : 752, Young 1990a : 227). Peru ( Young 1990a : 227). Surinam ( Young 1990a : 227). Trinidad ( Young 1990a : 227). Venezuela ( Young 1990a : 227). USA ( Young 1990a : 227).

***D. striga*** **Young, 1990b .** Brazil: Amazonas ( Young 1990b : 46), Pará ( Young 1990b : 46). Bolivia ( Young 1990b : 46). Peru (Nilsson 2001: 219, 2013: 199, Young 1990b : 46).

***D. strigata*** **Young, 1981.** Brazil: Mato Grosso do Sul ( Nilsson 2001 : 216, 2013: 197, Young 1981a : 64).

***D. subnotata*** **Zimmermann, 1921 .** Brazil: ( Trémouilles 1995 : 33, Young 1980: 319), Amazonas ( Braga & Ferreira-Jr 2010 : 40- 42), Mato Grosso do Sul ( Nilsson 2001 : 217, 2013: 197, Zimmermann 1921 : 192), Pará ( Braga & Ferreira-Jr 2010 : 40, 42).

***D. subtilis*** **Sharp, 1882.** Brazil: (Tré- mouilles 1995: 33, Young 1980 : 319), “Campos” ( Nilsson 2001 : 217, 2013: 197, Sharp 1882a : 341), Mato Grosso do Sul ( Zimmermann 1921 : 206). Argentina (Tré- mouilles 1995: 33).

Note: "Campos", in English fields, is a very common name and appears in hundreds of locations unfortunately without more information it is impossible to determine the correct one.

***D. suturalis*** **Sharp, 1882.** Brazil: (Tré- mouilles 1995: 31, Young 1980 : 310), “Santa Rita” ( Nilsson, 2001 : 216, 2013: 196, Sharp 1882a : 340). Argentina (Régimbart 1903: 47, Trémouilles 1995 : 31).

***D. ubangoides*** **Young, 1980 .** Brazil: (Tré- mouilles 1995: 32), Amazonas ( Nilsson 2001 : 218, 2013: 198, Young 1980 : 315). Ecuador ( Trémouilles 1995 : 32, Young 1980 : 315).

***D. undulatosterna*** **Braga & Ferreira, 2011 .** Brazil: Rio de Janeiro ( Braga & Ferreira-Jr 2011 : 130, Nilsson 2013 : 198).

***D. ukuki*** **sp. nov.** Brazil: Amazonas.

***D. varians*** **Wehncke, 1877 .** Brazil: (Tré- mouilles 1995: 31, Young 1980 : 310), Bahia ( Sharp 1882a : 341), Ceará ( Nilsson 2001 : 219, 2013: 199, Wehncke 1877 : 152).

***D. variegata*** **Sharp, 1882.** Brazil: (Tré- mouilles 1995: 32). El Salvador ( Young 1995 : 43). Guatemala ( Nilsson 2001 : 217, 2013: 198, Sharp, 1882b : 17, Young 1995 : 43). Honduras ( Young 1995 : 43). Mexico ( Nilsson 2001 : 217, Sharp, 1882b : 17, Young 1995 : 38, 42).

*D. varzeana* **Braga & Ferreira-Jr, 2010 .** Brazil: Amazonas ( Braga & Ferreira-Jr 2010 : 36, Nilsson 2013 : 196).

***D. volatidisca*** **Miller, 2001 .** Brazil: Minas Gerais (*), Rio de Janeiro (*). Bolivia (Miller 2001: 227, Nilsson 2001 : 216, 2013: 19).

***D. zelota*** **Young, 1990.** Brazil: Mato Grosso ( Nilsson 2001 : 217, 2013: 197, Young 1990b : 43), Mato Grosso do Sul ( Young 1990b : 43).

***D. phacoides*** **Guignot, 1950 .** Brazil: ( Young 1980 : 319). Bolivia ( Guignot 1950 : 2, Trémouilles 1995 : 33). Paraguay ( Nilsson 2001 : 216, 2013: 197, Guignot 1950 : 2, Trémouilles 1995 : 33).

Note: Young (1980) reported *D. phacoides* from Brazil, citing Guignot (1950 :1). Probably, this record is incorrect, because although Guignot mentioned on page 1 in his paper that the species studied here are from the Belgian Congo or South America (Brazil and Paraguay) ("Les espèces étudiées dans cette note proviennent soit du Congo Belge, soit de l 'Amérique du Sud (Brésil et Paraguay), et ..."), on page 2 are cited only Paraguay and Bolivia, without any indication of Brazil.

## Abbreviations

DZRJ: Entomological Collection Prof. José Alfredo Pinheiro Dutra, Departamento de Zoologia, Universidade Federal do Rio de Janeiro
INPA: Instituto Nacional de Pesquisas da Amazônia
MZSP: Museu de Zoologia da Universidade de São Paulo
